# Prevalence of genotype-specific human papillomavirus in cytology specimens and cervical biopsies, and its implication in cervical cancer risk stratification: a retrospective study of 10647 cases

**DOI:** 10.7150/jca.60601

**Published:** 2021-10-21

**Authors:** Feng Zhou, Yuezhou Chen, Amanda Louise Strickland, Hao Chen, Xiaofei Zhang

**Affiliations:** 1 Department of Pathology, Zhejiang University School of Medicine Women's Hospital, Hangzhou, Zhejiang Province, 310006, China.; 2 Reproductive Medicine Center, Zhongshan City People's Hospital, Zhongshan, Guangdong Province, 528403, China; 3 Department of Pathology, Northwestern University, Feinberg School of Medicine, Chicago, IL, 60611, USA.; 4 Department of Pathology, University of Texas Southwestern Medical Center, Dallas, TX, 75390, USA.

**Keywords:** Risk stratification, Cervical screening, Cancer, Human papillomavirus, E6/E7 mRNA genotyping

## Abstract

**Objective:** This study aimed to describe the risk stratification of squamous cell carcinoma (SCC) and its precursor lesions based on HPV E6/E7 mRNA genotyping.

**Methods:** 10647 hrHPV+ women (mean age 40.8 years), who had concurrent cytology and follow-up biopsy results available between September 2016 and May 2020, met the inclusion criteria and were selected for immediate risk analysis.

**Results:** In this cohort, HPV-16 or 18/45+ women had significantly higher immediate risk of cervical cancer and precancer compared with other genotypes+ women. The relative immediate risk (RIR) of ASC-H+ was 2.0 (95% CI: 1.9-2.4) and SCC was 9.4 (95% CI: 5.5-15.6) for HPV-16 or 18/45+ women when compared with women positive for other 11 genotypes. Among follow-up biopsy cases, the RIR of CIN2+ was 2.7 (95% CI: 3.0-3.7) and SCC was 10.8 (95% CI: 7.2-17.4) for HPV-16 or 18/45+ women than women positive for other genotypes. Similarly, when compared with women positive for other genotypes, the RIR of CIN2+ was 2.9 (95% CI: 2.7-4.6) and SCC was 13.8 (95% CI: 3.0-66.2) for HPV-16 or 18/45+ women with ASC-US, and RIR of CIN2+ was 3.3 (95% CI: 3.1-4.6) and SCC was 22.3 (95% CI: 2.8-176.8) for HPV-16 or 18/45+ women with NILM.

**Conclusions:** This study supports that hrHPV mRNA genotyping can be an effective risk stratification tool to identify individual at higher risk for cervical cancer or precancer, and provides important evidences for the future modifications for current China cervical cancer screening guidelines.

## Introduction

The vast majority of cervical cancer cases are associated with infection by high risk human papillomavirus (hrHPV), the most common viral infection of the reproductive tract worldwide 1. Persistent hrHPV infection is the primary cause of cervical cancer and its precursor lesion, high-grade cervical intraepithelial neoplasia (CIN2+) 2-4.

The incidence and mortality of cervical cancer have largely decreased due to the implementation of preventive cervical cancer screening programs. However, there is still ongoing debate about the best model for routine cervical cancer screening. Consensus guidelines for management of cervical dysplasia in the screening setting have not yet been reached to accommodate the three most widely available screening strategies: primary HPV testing, co-testing with HPV testing and cervical cytology, and cervical cytology 5. But cervical cytology has been proven to have low sensitivity for high-grade lesions; therefore, multiple tests must be performed for each case 6. HPV DNA tests, such as the Hybrid Capture 2 assay (HC2) HPV and Cobas 4800 HPV, have been used separately as primary cervical cancer screening tools in women aged 25 years or older in many countries 7-9. However, developing such guidelines is complex because HPV infections are transient and therefore sometimes clinically insignificant. But because it is unknown which patients will clear the virus and indeed have transient infections, all positive cases warrant further workup. This “universal” style of HPV DNA testing could therefore result in increased costs and patient anxiety 10.

The hrHPV randomly integrates into the host genome through a process that includes the disruption of some part of E2 gene which in turn causes the overexpression of the E6 and E7 proteins 11, 12. Overexpression of E6 and E7 can alter various signaling pathways related to cellular transformation and immune escape, which is a crucial step for carcinogenesis 13. Many new studies propose HPV E6/E7 mRNA testing as a more specific test compared to other methods such as HPV DNA, repeated cytology, and colposcopy for the follow-up of women with borderline findings, such as atypical squamous cells of unknown significance (ASC-US) 14-16. In addition, HPV E6/E7 mRNA testing may be more useful as a screening test for early detection and prediction of subsequent progression to severe dysplasia 17-19. Oncogenic HPV genotypes including 16, 18, 31, 33, 39, 45, 51, 52, 56, 58, 59, 66, and 68 are detected in over 99.7% of cervical cancer cases 20. Additionally, the hrHPV E6/E7 mRNA test has been proven to be a promising non-invasive biomarker for the detection of CIN2+, and at the same time allows detection of the HPV infection and prediction of progression of dysplasia 21-24. Although, several large scale studies such as POASCAN and ATHENA trials has investigated the HPV genotype prevalence and associated cervical cancer risk stratification 25, 26, those studies were mostly conducted in western countries where Caucasian women consist majority of the population. However, only limited large-scale population studies were conducted in Chinese population. Moreover, considering the size, social-economic, ethnic diversity of Chinese population, such large-scale study is much needed.

The aim of this study is to describe the risk stratification of squamous cell carcinoma (SCC) and its precursor lesions in this southern Chinese female population based on HPV genotyping by the Aptima human papillomavirus (AHPV) assay. This test modality was performed on patients referred from routine screening; these referred patients underwent cytological, AHPV assay and biopsy and/or curettage examination. Our study is, to our knowledge and so far, the largest single center this type study in China.

## Materials and methods

### Study design

**Figure 1:**
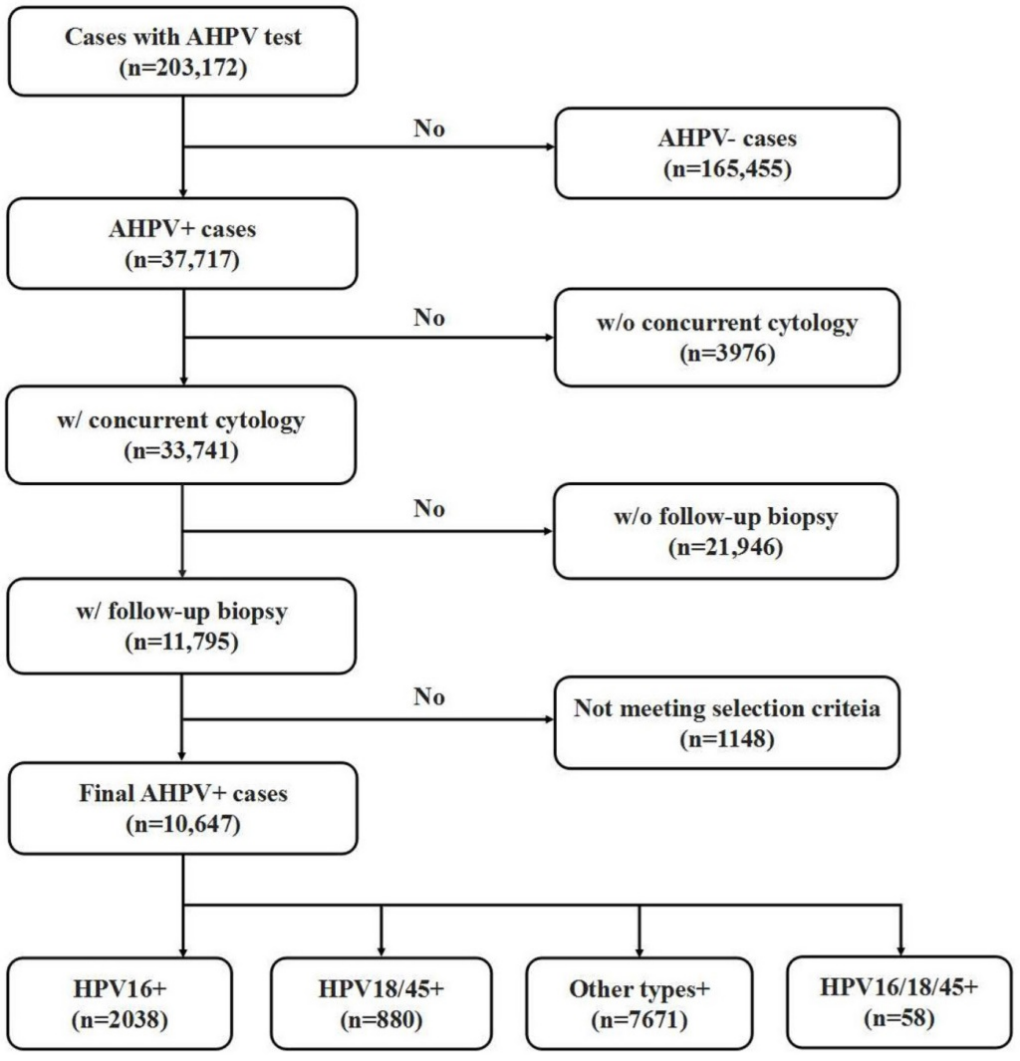
Flow chart of selection criteria of participants

This study was approved by the Zhejiang University School of Medicine Women's Hospital Institutional Review Board. A retrospective, computer-based search in the clinical information system database at the Zhejiang University School of Medicine Women's Hospital was performed to identify cases that underwent the AHPV assay between September 2016 to May 2020. As one of the biggest women hospitals in China, Women's Hospital provides care of over 1,500,000 outpatients & 77,000 inpatients annually. Women's hospital is also a cervical cancer screen center of Zhejiang province, one of the most developed and populated provinces of China. Patients included in current study represent women population of an east coast province of southern China who underwent routine clinical cervical cancer screening, with a mixture of urban and rural women population. As shown in Fig 1, a total of 203,172 women with AHPV assay results were identified. Among them, 36,717 AHPV+ women were identified, and 33,741 AHPV+ women had concurrent cytology results. Among those AHPV+ women who also had concurrent cytology results, 11,795 had undergone follow-up colposcopy examination with biopsy and/or curettage based on the ASCCP guidelines 5, 27. Because endocervical curettage (ECC) has been increasingly incorporated in the colposcopy-biopsy examination for women undergoing colposcopic evaluation 28-30, in our institution, women with abnormal colposcopy finding underwent lesion-targeted biopsy. Generally, if the colposcopy examination was unsatisfactory (the squamocolumnar junction was not completely visible), ECC was performed. Colposcopists were made aware of the cytology and AHPV results before the colposcopy visit was performed. The lack of histologic follow-up results of the rest of patients are due to the patients being lost to follow up or receiving care elsewhere. In summary, 10, 647 of AHPV+ cases met the inclusion criteria and were selected for further analysis. Patient ages in the final cohort ranged from 17 to 83 years (mean age 40.8 years). Inclusion criteria were: 1) No previously confirmed intraepithelial neoplasia, cervical cancer, or other malignancies; 2) No history of therapeutic cervical procedures, such as cervical microwave, electrocautery and other physical treatment or cervical conization; 3) Cases with abnormal glandular lesions were excluded from this study because the natural histories of adenocarcinoma (ADC) and adenocarcinoma in situ (AIS) differ from those of SCC and its precursor lesions.

### Liquid-based cytology

A single exfoliated cervical specimen was collected from each participant using a routinely available collection device, which was then rinsed immediately into the ThinPrep (PreservCyt® Solution, Hologic, Inc., Marlborough, MA, USA) container for AHPV and the liquid-based cytology (LBC) assay. Cytology slides were produced automatically by Thin Prep 2000 (Cytyc Corporation, Marlborough, MA). The LBC slides were classified according to the 2014 Bethesda System (TBS) into the following categories: 1) negative for intraepithelial lesion or malignancy (NILM); 2) atypical squamous cells of unknown significance (ASC-US); 3) low-grade squamous intraepithelial lesions (LSIL); 4) atypical squamous cells, cannot exclude high-grade squamous intraepithelial lesion (ASC-H); 5) high-grade squamous intraepithelial lesions (HSIL); 6) squamous cell carcinoma (SCC). ASC-H+ was defined as ASC-H or higher (including HSIL and SCC).

### HPV E6/E7 mRNA genotyping

Residual LBC samples were processed for the AHPV assay, according to the manufacturer's specifications. The AHPV and AHPV-GT (Gen-Probe Inc., San Diego, CA) were performed using an automated Panther System (Hologic, Inc., San Diego, CA). The E6/E7 oncogenic mRNA, which is associated with 14 hrHPV genotypes (16, 18, 31, 33, 35, 39, 45, 51, 52, 56, 58, 59, 66, and 68), was detected by the AHPV. AHPV+ samples were reflex-tested with the AHPV-GT, which can detect the HPVE6/E7 mRNA in hrHPV genotypes 16 and 18/45 or both. AHPV-GT-negative meant the other 11 hrHPV genotypes were positive. Patients who had indeterminate results or residual samples with quantities insufficient for hrHPV assay were excluded from the current study.

### Histologic diagnosis

Histologic correlation results including cervical biopsy and ECC performed within 6 months of the Pap and AHPV assay were included in this study. Histologic results were categorized into three general groups: 1) benign, 2) low-grade cervical lesion (CIN1), and 3) high-grade cervical lesion (CIN2+), defined as CIN2 or higher (CIN3 and SCC). Histopathologic diagnoses were rendered by subspecialized gynecologic pathologists. Cases initially diagnosed as CIN2/3 were confirmed by a second reviewing pathologist for quality assurance purposes. Immunohistochemical staining with p16 and Ki-67 was also liberally used to support the CIN2/3 diagnoses. In patients with more than one tissue sample, the highest grade diagnosis was recorded 31.

### Statistical analysis

To analyze the risk of ASC-H+ in cytology specimens, cytology results of ASC-H, HSIL, and SCC were combined into a single “high-grade” category. Meanwhile, NILM, ASC-US and LSIL were combined into a single “non-high-grade” category. The cases were categorized according to the HPV genotypes. Similarly, cytology diagnoses were combined into non-SCC and SCC categories. The two-tailed chi-square test was used to compare the prevalence of “high-grade” lesions or SCC among HPV genotype groups.

To analyze the risk of CIN2+ lesions in biopsy specimens, histologic results of CIN2, CIN3 and SCC were combined into a single “high-grade” category, while normal and CIN1 were combined into a single “non-high-grade” category. The cases were categorized according to the HPV genotypes. Similarly, diagnoses were combined into non-SCC and SCC categories. The two-tailed chi-square test was used to compare the prevalence of “high-grade” lesions or SCC among HPV genotype groups. The same strategy was used to analyze the risk of “high-grade” lesions in follow-up biopsies from patients with cytology diagnoses of ASC-US. Statistical analyses were performed using JMP 11.2.0 (SAS Institute Inc., Cary, NC). A significance level of 0.05 was used.

## Results

### Specific HPV genotype prevalence per cytologic diagnosis among hrHPV+ women

**Figure 2:**
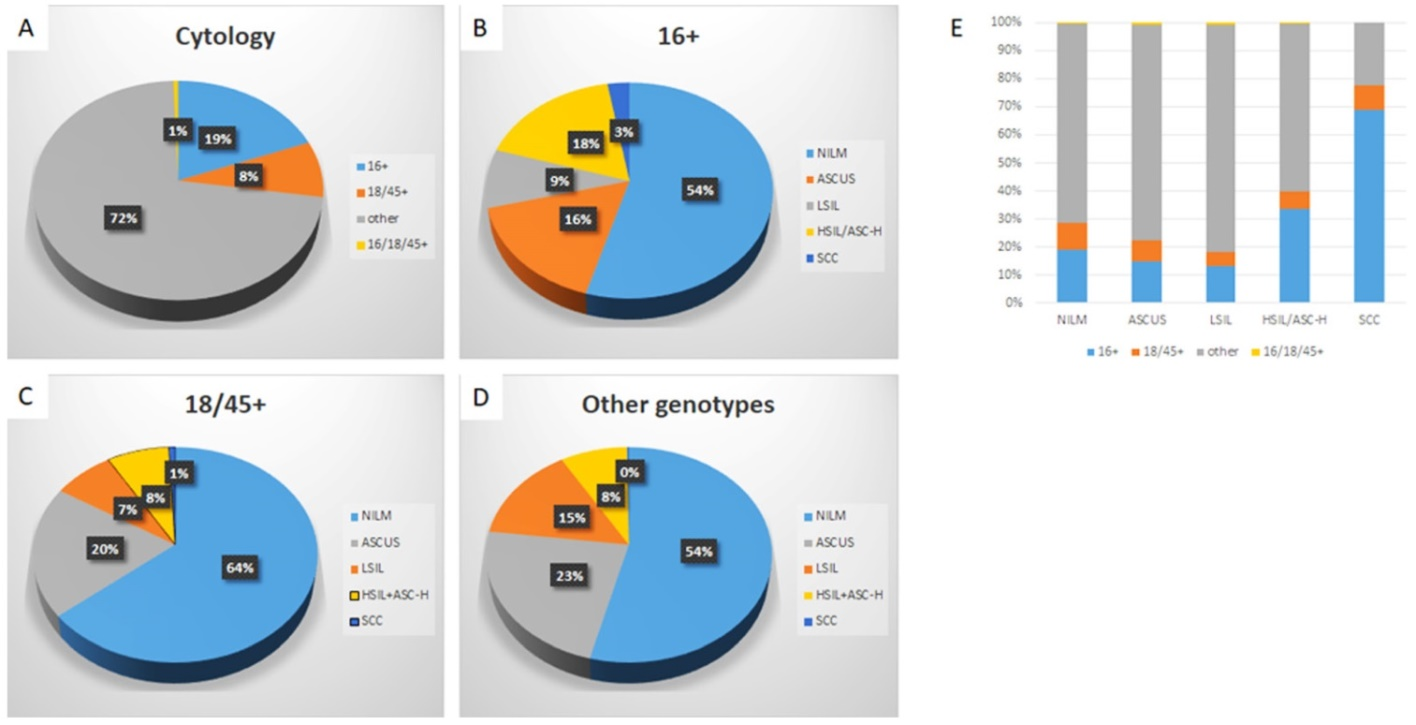
** Distribution of HPV genotypes in hrHPV+ women according to cytology diagnosis.** The distribution of HPV genotypes in hrHPV+ women (A); The distribution of cytologic diagnoses in HPV-16+ (B), HPV-18/45+ (C), and the other genotypes+ women (D); The genotype distribution in individual cytological diagnosis (E). The trend of increasing prevalence of HPV-16 in ASC-H+ lesions is significant, p (<.05).

Detailed analysis is included in Table 1, 2 and Figure 2. Among hrHPV+ cytology cases, 19.1% were positive for HPV-16, 8.3% were positive for HPV-18/45, 72.0% were positive for other 11 genotypes, and 0.5% positive for both HPV-16 and HPV-18/45. The distribution of cytologic diagnoses in hrHPV+ cytology cases is as follows: 55.0% as NILM, 21.5% as ASC-US, 12.8% as LSIL, 10.0% as ASC-H or HSIL, and 0.8% as SCC.

When further analyzed along the genotypes, genotype-specific risk stratification of ASC-H+ cytology was observed. 17.5% HPV-16+ women were diagnosed as ASC-H/HSIL and 2.7% as SCC, while 7.7% and 0.8% HPV-18/45+ women and 8.3% and 0.2% other genotype+ women were diagnosed as ASC-H/HSIL and SCC respectively. In this cohort, when compared to women with infection by other genotype groups, HPV-16+ women had significantly higher immediate risk of ASC-H+ (p<0.00001 16+ vs 18/45+; p<0.00001 16+ vs other genotypes+) and SCC (p=0.0011 16+ vs 18/45+; p<0.00001 16+ vs other genotypes+). In addition, HPV-18/45+ women in this cohort showed significantly higher immediate risk of SCC than women positive for other 11 genotypes (p=0.0035). The relative immediate risk (RIR) of ASC-H+ was 2.4 (95% CI: 2.4-3.1) and SCC was 11.5 (95% CI: 6.9-20.1) for HPV-16+ women when compared with women positive for other 11 genotypes. Together RIR of ASC-H+ was 2.0 (95% CI: 1.9-2.4) and SCC was 9.4 (95% CI: 5.5-15.6) for HPV-16 or 18/45+ women when compared with women positive for other 11 genotypes.

Despite the relatively low prevalence among hrHPV+ cytology cases (19.1%), HPV-16 accounted for 33.6% of subsequent HSIL and 68.8% of SCC. HPV-18/45 accounted for 6.3% of ASC-H/HSIL and 8.8% of SCC. Meanwhile, the other 11 genotypes accounted for 59.7% ASC-H/HSIL and 22.5 % of SCC.

### Specific HPV genotype prevalence per histologic diagnosis among hrHPV+ women

**Figure 3:**
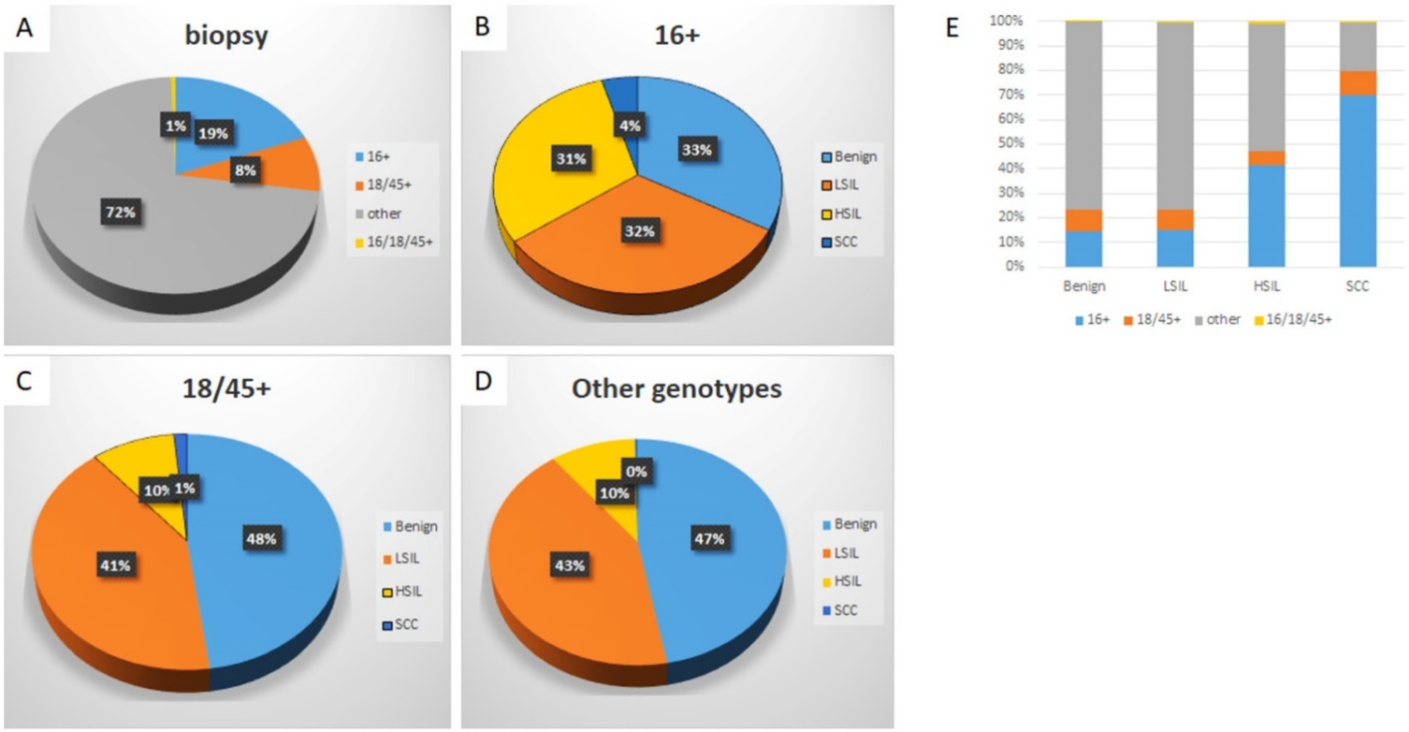
** Distribution of HPV genotypes in hrHPV+ women according to histologic diagnosis.** The distribution of HPV genotypes in hrHPV+ women (A); The distribution of histologic diagnoses in HPV-16+ (B), HPV-18/45+ (C), and other genotypes+ women (D); The genotype distribution in individual histological diagnosis (E). The trend of increasing prevalence of HPV-16 in CIN2+ lesions is significant, p (<.05).

Detailed analysis is included in Table 1, 3 and Figure 3. The distribution of histologic diagnoses in hrHPV+ biopsies is as follows: 44.4% as benign, 40.3% as LSIL, 14.1% as HSIL (including CIN2 and CIN3), and 1.2% as SCC. When further analyzed along the genotypes, genotype-specific risk stratification of CIN2+ was observed. 30.6% HPV-16+ women were diagnosed as HSIL and 4.4% as SCC, while 9.5% and 1.5% HPV-18/45+ women and 10.1% and 0.3% other 11 genotype+ women were diagnosed as HSIL and SCC respectively. In this cohort, HPV-16+ women had significantly higher immediate risk of CIN2+ lesions (p<0.00001 16+ vs 18/45+; p<0.00001 16+ vs other genotypes+) and SCC (p=0.000079 16+ vs 18/45+; p<0.00001 16+ vs other genotypes+) than women in other genotype groups. In addition, HPV-18/45+ women showed significantly higher risk for SCC than women positive for other 11 genotypes (p<0.00001). The RIR of CIN2+ was 3.4 (95% CI: 4.1-5.2) and SCC was 13.6 (95% CI: 9.0-22.1) for HPV-16+ women when compared with women positive for other 11 genotypes. Together RIR of CIN2+ was 2.7 (95% CI: 3.0-3.7) and SCC was 10.8 (95% CI: 7.2-17.4) for HPV-16 or 18/45+ women when compared with women positive for other 11 genotypes. Although not statistically significant due to small power (n=58), HPV 16 and 18/45 dual positive women (n=58) has a similar risk of HSIL and SCC as HPV-16+ women, with 36.2% diagnosed as HSIL and 1.7% as SCC.

Despite the relatively low prevalence among hrHPV+ cases (19.1%), HPV-16 accounted for 41.5% of HSIL and 69.8% of SCC. HPV-18/45 accounted for 5.6% of HSIL and 10.1 % of SCC and other 11 genotypes accounted for 51.5% HSIL and 19.4% of SCC.

### Specific HPV genotype prevalence per histologic diagnosis among hrHPV+ women with ASC-US

Detailed analysis is included in Table 4. Among all hrHPV+ cases with ASC-US, 14.7% were positive for HPV-16, 7.7% were positive for HPV-18/45, 77.0% were positive for other 11 genotypes, and 0.7% positive for both HPV-16 and HPV-18/45. The prevalence of CIN2+ and SCC was 12.2% and 0.4%, respectively. Among HPV-16+ ASC-US cases, 31.9% were diagnosed as CIN2+ and 1.8% were diagnosed as SCC in follow-up cervical biopsies. Among HPV-18/45+ ASC-US cases, 9.8% were diagnosed as CIN2+ and 1.2% were diagnosed as SCC in follow-up cervical biopsies. In contrast, among ASC-US cases positive for other 11 genotypes, 8.4% were diagnosed as CIN2+ and 0.1% were diagnosed as SCC in follow-up cervical biopsies.

Despite the relatively low prevalence in hrHPV+ women with ASC-US (14.7%), HPV-16 accounted for 38.4% of HSIL and 60% of SCC in follow-up biopsies. HPV-18/45 and other 11 genotypes accounted for 6.1% and 53.0% of CIN2+ lesion respectively, while both genotype groups accounted for 20% of all SCC, respectively. Although there was no significant difference in immediate risk of CIN2+ lesions between HPV-18/45 and other 11 genotypes (p=0.28), the immediate risk of SCC in follow-up biopsies was significantly higher in HPV-18/45+ women than other 11 genotypes positive women (p=0.004). The RIR of CIN2+ was 3.8 (95% CI: 3.8-6.8) and SCC was 15.7 (95% CI: 3.2-79.5) for HPV-16+ women with ASC-US when compared with women positive for other 11 genotypes. Together RIR of CIN2+ was 2.9 (95% CI: 2.7-4.6) and SCC was 13.8 (95% CI: 3.0-66.2) for HPV-16 or 18/45+ women when compared with women positive for other 11 genotypes.

### Specific HPV genotype prevalence per histologic diagnosis among hrHPV+ women with NILM

Detailed analysis is included in Table 5. Among all hrHPV+ cases with NILM, 19.0% were positive for HPV-16, 9.7% were positive for HPV-18/45, 70.8% were positive for hrHPV genotypes other than 16, 18 and 45, and 0.5% positive for both HPV-16 and HPV-18/45. The prevalence of CIN2+ and SCC was 7.7% and 0.2%, respectively. Among HPV-16+ cases, 19.4% were diagnosed as CIN2+ and 0.7% were diagnosed as SCC in follow-up cervical biopsies. Among HPV-18/45+ cases, 6.7% were diagnosed as CIN2+ and 0.2% were diagnosed as SCC in follow-up cervical biopsies. In contrast, among cases positive for genotypes other than 16 or 18/45, 4.6% were diagnosed as CIN2+ and 0.02% were diagnosed as SCC in follow-up cervical biopsies.

Despite the relatively low prevalence in women hrHPV+ with NILM (19.0%), HPV-16 accounted for 47.5% of HSIL and 80% of SCC in follow-up biopsies. HPV-18/45 accounted for 8.4% of CIN2+ and 10% of SCC and other genotypes accounted for 42.9% HSIL and 10% of SCC in follow-up biopsies.

Although the immediate risk of CIN2+ and SCC in hrHPV+ women with NILM was significantly lower than hrHPV+ women with ASC-US cytology (p<0.00001 for CIN2+, and p=0.03 for SCC), there was still a significant immediate risk of CIN2+ lesion in women who were hrHPV+ with NILM (7.7%), especially among HPV-16+ women (19.4%). The RIR of CIN2+ was 4.3 (95% CI: 4.1-6.2) and SCC was 29.9 (95% CI: 3.8-240.6) for HPV-16+ women when compared with women positive for other 11 genotypes. Together RIR of CIN2+ was 3.3 (95% CI: 3.1-4.6) and SCC was 22.3 (95% CI: 2.8-176.8) for HPV-16 or 18/45+ women when compared with women positive for other 11 genotypes.

## Discussion

The prevalence of HPV infection and genotype-specific distribution vary greatly among nations 32, and even among different regions within countries 33. A nationwide study in China showed the most prevalent genotypes were HPV-16 and HPV-52, followed by HPV-58, and HPV-18 was the seventh most common infection 33. In this study, HPV-16 accounts for 19.1%, HPV-18/45 accounts for 8.2%, and the other 11 genotypes account for 72.0% of hrHPV+ cases.

In hrHPV+ cytology cases, genotype-specific risk stratification of ASC-H+ cytology was observed in 17.5% of HPV-16+ women diagnosed as ASC-H or HSIL and 2.7% as SCC, while 7.7% and 0.8% HPV-18/45+ women and 8.3% and 0.2% other 11 genotype+ women were diagnosed as ASC-H or HSIL and SCC, respectively. HPV-16+ women had a significantly higher immediate risk of ASC-H+ and SCC than women with infection by the other 11 genotype groups. Despite the relatively low prevalence, HPV-16 and 18/45 accounted for 40% of all ASC-H and HSIL cases, and 77.5% of all SCC cases with HPV-16 alone accounting for 69.8% of all SCC cases. Our data is comparable to that of a Danish study which showed an increased prevalence of HPV-16 and 18 in more severely abnormal cytology findings, and implemented HPV-16 as the genotype most frequently associated with SCC (61.6%) 34.

Similar risk stratification was observed in hrHPV+ biopsy cases, such that HPV-16 or 18/45+ women have significantly higher immediate risk of CIN2+ and SCC than women infected with other high-risk genotypes. In addition, HPV-16 and 18/45 accounted for 47.1% of all CIN2+, and 79.8% of all SCC with HPV-16 alone accounting for 69.8% of all SCC. Thus, we are providing further evidence that persistent hrHPV infection is the primary cause of high-grade cervical disease, and persistent infection with HPV-16 and 18/45 pose higher risk for CIN2+ and SCC compared to other high-risk genotypes 31, 35-37. Our findings are consistent with those findings in the POBASCAN and ATHENA trials 25, 26, both of which demonstrated higher risk for precancer and cancer in HPV-16/18+ women than women positive for other high-risk genotypes. The Portland-Kaiser study also found that HPV-16 positivity had 2.7 times higher risk of precancer and cancer than other high-risk genotypes across all cytology results 38.

A pooled hrHPV assay has been routinely used worldwide to stratify risks in women with ASC-US 31. The ATHENA study demonstrated that HPV genotyping may be a promising risk stratification tool in aiding management decision-making for women with ASC-US 36. In our study, the overall prevalence of CIN2+ and SCC was 12.2% and 0.4% respectively, which is comparable to that of other large-scale studies 39, 40. Furthermore, 31.8% of HPV-16+ women with ASC-US were diagnosed as CIN2+ and 1.8% as SCC in follow-up cervical biopsies. These risks are significantly higher than women positive for the other 11 genotypes. In addition, HPV-18/45+ women with ASC-US also showed significantly higher risk of SCC than women positive for high-risk genotypes other than 16 or 18/45.

The hrHPV-positive, cytology-negative (NILM) category has been a special challenge for patient management ever since cytology and HPV co-assay was approved by the Food and Drug Administration (FDA) in 2003 37. Similar to that of other studies 39, 40, the prevalence of CIN2+ and SCC was 7.7% and 0.2% respectively in current study. Although the overall prevalence of CIN2+ lesions and SCC is significantly lower in women hrHPV+ with NILM than that of women hrHPV+ with ASC-US, similar risk stratification of CIN2+ lesion and SCC is noted among genotypes, with HPV-16+ women showing the highest immediate risk for CIN2+ lesions (19.4%) and SCC (0.7%) in follow-up biopsies. In contrast, only 4.6% and 0.02% of women positive for high-risk genotypes other than 16 or 18/45 were diagnosed of CIN2+ lesion and SCC in follow-up cervical biopsies, respectively. Our data is similar to that of a previous study by Han et al 37, in which 11.5% of HPV-16 or 18/45+ women and 3.6% of women positive for other genotypes were diagnosed with CIN2+ lesions in follow-up biopsies. Additionally, we identified 58 women with dual positivity for HPV-16 and -18/45; this sub-cohort had a similar distribution of histologic diagnoses as did the HPV16+ women. Whether a competitive or cooperative interaction exists among the co-infecting HPV genotypes is still unclear. A recent study found co-infections of HPV-16 and other hrHPVs reduced the incidence of CIN3+ 41. In our study, no competitive effect was observed. A larger sample size of women positive for multiple hrHPV genotypes may help provide a more definitive answer to the possibility of competitive effect of hrHPVs. Overall, our study provides a “snapshot” of prevalence of hrHPV genotypes, the distribution of cervical lesions and a general assessment of the genotype specific immediate risk of cancer and precancer in Women of Zhejiang province. Studied populations represent a women population from an east coast province of China with a mixture of urban and rural population.

There were several limitations in current study. First, as a tertiary referral center, vast majority of our patients got their follow-up and/or treatment at lower-level regional hospital after initial diagnosis. Therefore, we were not able to collect complete long-term follow-up information for most of patients involved in this study. Thus, our study only provided a general assessment of the immediate risk of SCC and its precursor lesions by HPV genotypes in this southern Chinese women population. Future large studies that include hrHPV+ women with sufficient and long-term follow-up information to validate the cervical cancer risk stratification results within this population. However, due to still lack of national/or regional Cervical Cancer Screening Registration Program, the long-term risk analysis in Chinese population is yet to be conducted. This further emphasizes the urgency of establishing such program in China. Second, those cases with abnormal glandular lesions such as ADC and AIS were excluded from this study because the natural history and underlying mechanism of ADC and AIS differ from those of SCC and its precursor lesions. The relationship between specific HPV genotyping and abnormal glandular lesions will be addressed separately in a future study. But to specifically address the risk stratification of SCC and its precursor lesions based on HPV genotyping, the large volume of included cases will offer higher statistical power.

There are limited large scale studies on HPV genotype-based cervical cancer risk stratification in the Chinese female population 42. The utility of HPV genotyping has not been clearly defined in current Chinese cervical cancer screening guidelines 43. Our study is, to our knowledge and so far, the largest single center study to include hrHPV genotyping, cytology and biopsy studies on hrHPV+ women. Our data support the argument that HPV genotyping can be an effective risk stratification tool 21-24 and can potentially help reduce overall referral for colposcopy and biopsy, especially for women positive for genotypes other than 16 or 18/45 with ASC-US/NILM. On the other hand, surveillance alone is likely to be too risky for HPV-16 or 18/45+ women with ASC-US/NILM. Moreover, our results support that Aptima assay separately can be as a screening test for early detection and prediction of cancer and precancer, and provides important evidence for future modification of current national guidelines for Chinese women.

## Figures and Tables

**Table 1:** The immediate risk of cervical cancer and precancer in hrHPV+ women

hrHPV genotype	Cytologic diagnoses		Histologic diagnoses		
	ASC-H+	SCC	CIN2+	SCC	Total
16+	412(20.2%)	55 (2.7%)	713 (35.0%)	90 (4.4%)	2038
18/45+	74 (8.4%)	7 (0.8%)	97 (11.0%)	13 (1.5%)	880
other+	653 (8.5%)	18 (0.2%)	799 (10.4%)	25 (0.3%)	7671
P value	<0.00001, 16+ vs 18/45+	0.0011, 16+ vs 18/45+	<0.00001, 16+ vs 18/45+	0.000079, 16+ vs 18/45+	
	<0.00001, 16+ vs other +	<0.00001, 16+ vs other +	<0.00001, 16+ vs other +	<0.00001, 16+ vs other+	
		0.0035, 18/45+ vs other +		<0.00001, 18/45+ vs other +	

Abbreviations: hrHPV+, high-risk human papillomavirus positivity; HSIL, high-grade squamous intraepithelial lesion; ASC-H, atypical squamous cells that could not exclude HSIL; SCC, squamous cell carcinoma; CIN1: cervical intraepithelial neoplasia Grade 1; CIN2: cervical intraepithelial neoplasia Grade 2; CIN3: cervical intraepithelial neoplasia Grade 3; ASC-H+ was defined as ASC-H or higher (including HSIL and SCC)); CIN2+, defined as CIN2 or higher (including CIN3 and SCC).

**Table 2:** Genotype-specific distribution of cytologic diagnoses in hrHPV+ women

Cytologic diagnosis	hrHPV genotype				
	16+	18/45+	Other+	16+, 18/45+	Total
NILM	1111 (19.0%)	565 (9.7%)	4145 (70.8%)	30 (0.5%)	5851
ASCUS	336 (14.7%)	174 (7.6%)	1760 (77.0%)	16 (0.7%)	2286
LSIL	179 (13.1%)	67 (4.9%)	1113 (81.4%)	8 (0.6%)	1367
ASC-H/HSIL	357 (33.6%)	67 (6.3%)	635 (59.7%)	4 (0.4%)	1063
SCC	55 (68.8%)	7 (8.8%)	18 (22.5%)	0 (0.0%)	80
Total	2038 (19.1%)	880 (8.3%)	7671 (72.0%)	58 (0.5%)	10647

Abbreviations: NILM: negative for intraepithelial lesion or malignancy; ASC-US, atypical squamous cells of undetermined significance; LSIL, low-grade squamous intraepithelial lesions; HSIL, high-grade squamous intraepithelial lesion; ASC-H, atypical squamous cells that could not exclude HSIL; SCC, squamous cell carcinoma; hrHPV+, high-risk human papillomavirus positivity.

**Table 3:** Genotype-specific distribution of histologic diagnoses among hrHPV+ women

Histologic diagnosis	hrHPV genotype				
	16+	18/45+	Other+	16+, 18/45+	Total
Benign	676 (14.3%)	421 (8.9%)	3612 (76.5%)	15 (0.3%)	4724
CIN1	649 (15.1%)	362 (8.4%)	3260 (76.0%)	21 (0.5%)	4292
CIN2	201 (32.0%)	46 (7.3%)	377 (60.0%)	5 (0.8%)	629
CIN3	422 (48.3%)	38 (4.4%)	397 (45.5%)	16 (1.8%)	873
SCC	90 (69.8%)	13 (10.1%)	25 (19.4%)	1 (0.8%)	129
Total	2038 (19.1%)	880 (8.3%)	7671 (72.0%)	58 (0.5%)	10647

Abbreviations: CIN1: cervical intraepithelial neoplasia Grade 1; CIN2: cervical intraepithelial neoplasia Grade 2; CIN3: cervical intraepithelial neoplasia Grade 3; SCC, squamous cell carcinoma;hrHPV+, high-risk human papillomavirus positivity.

**Table 4:** F/U biopsy diagnoses in women hrHPV+ with ASC-US cytology

Histologic diagnosis	hrHPV genotype				
	16+	18/45+	Other+	16+, 18/45+	Total
Benign	74 (9.1%)	60 (7.4%)	673 (83.1%)	3 (0.4%)	810
CIN1	155 (12.9%)	97 (8.1%)	939 (78.4%)	6 (0.5%)	1197
CIN2	43 (31.6%)	10 (7.4%)	81 (59.6%)	2 (1.5%)	136
CIN3	58 (43.6%)	5 (3.8%)	65 (48.9%)	5 (3.8%)	133
SCC	6 (60.0%)	2 (20.0%)	2 (20.0%)	0 (0.0%)	10
Total	336 (14.7%)	174 (7.6%)	1760 (77.0%)	16 (0.7%)	2286

Abbreviations: CIN1: cervical intraepithelial neoplasia Grade 1; CIN2: cervical intraepithelial neoplasia Grade 2; CIN3: cervical intraepithelial neoplasia Grade 3; SCC, squamous cell carcinoma; hrHPV+, high-risk human papillomavirus positivity.

**Table 5:** F/U biopsy diagnoses in women hrHPV+ with NILM cytology

Histologic diagnosis	hrHPV genotype				
	16+	18/45+	Other+	16+, 18/45+	Total
Benign	540 (15.6%)	334 (9.7%)	2572 (74.4%)	11 (0.3%)	3457
CIN1	355 (18.2%)	193 (9.9%)	1384 (71.1%)	14 (0.7%)	1946
CIN2	87 (4.4%)	22 (1.1%)	113 (5.8%)	2 (0.1%)	224
CIN3	121 (56.5%)	15 (7.0%)	75 (35.0%)	3 (1.4%)	214
SCC	8 (80.0%)	1 (10.0%)	1 (10.0%)	0 (0.0%)	10
Total	1111 (19.0%)	565 (9.7%)	4145 (70.8%)	30 (0.5%)	5851

Abbreviations: CIN1: cervical intraepithelial neoplasia Grade 1; CIN2: cervical intraepithelial neoplasia Grade 2; CIN3: cervical intraepithelial neoplasia Grade 3; SCC, squamous cell carcinoma; hrHPV+, high-risk human papillomavirus positivity.

## Abbreviations

AHPV: Aptima human papillomavirus
ECC: endocervical curettage
ADC: adenocarcinoma
AIS: adenocarcinoma in situ
NILM: negative for intraepithelial lesion or malignancy
ASC-US: atypical squamous cells of undetermined significance
LSIL: low-grade squamous intraepithelial lesions
HSIL: high-grade squamous intraepithelial lesion
ASC-H: atypical squamous cells that could not exclude HSIL
SCC: squamous cell carcinoma
CIN1: cervical intraepithelial neoplasia Grade 1
CIN2: cervical intraepithelial neoplasia Grade 2
CIN3: cervical intraepithelial neoplasia Grade 3
SCC: squamous cell carcinoma
RIR: relative immediate risk
hrHPV+: high-risk human papillomavirus positivity
